# Metabolic profile in patients with newly diagnosed bipolar disorder and their unaffected first-degree relatives

**DOI:** 10.1186/s40345-019-0142-3

**Published:** 2019-04-02

**Authors:** Klara Coello, Maj Vinberg, Filip K. Knop, Bente K. Pedersen, Roger S. McIntyre, Lars V. Kessing, Klaus Munkholm

**Affiliations:** 1 Copenhagen Affective Disorders Research Centre (CADIC), Psychiatric Center Copenhagen, Department O, 6243, Rigshospitalet, Blegdamsvej 9, 2100 Copenhagen, Denmark; 2 Department of Clinical Medicine, Faculty of Health and Medical Sciences, University of Copenhagen, Copenhagen, Denmark; 3 Clinical Metabolic Physiology, Steno Diabetes Center Copenhagen, Gentofte Hospital, Hellerup, Denmark; 4 Centre of Inflammation and Metabolism (CIM) and the Centre for Physical Activity Research (CFAS), Rigshospitalet, Copenhagen, Denmark; 5 Mood Disorders Psychopharmacology Unit, University Health Network, Toronto, Canada

**Keywords:** Bipolar disorder, Insulin resistance, Metabolic syndrome X, Co-morbidity

## Abstract

**Objective:**

The prevalence of metabolic syndrome and insulin resistance is twice as high in patients with bipolar disorder compared with the general population, and possibly associated with a disabling illness trajectory of bipolar disorder, an increased risk of cardiovascular disease and premature death. Despite these detrimental effects, the prevalence of metabolic syndrome and insulin resistance in patients newly diagnosed with bipolar disorder and their unaffected first-degree relatives is largely unknown.

**Methods:**

In a cross-sectional study of 206 patients with newly diagnosed bipolar disorder, 50 of their unaffected first-degree relatives and 109 healthy age- and sex-matched individuals, we compared the prevalence of metabolic syndrome and insulin resistance (HOMA-IR). In patients with bipolar disorder, we further investigated illness and medication variables associated with the metabolic syndrome and insulin resistance.

**Results:**

Higher rates of metabolic syndrome (odds ratio = 3.529, 95% CI 1.378–9.041, *P *= 0.009) and levels of insulin resistance (B = 1.203, 95% CI 1.059–1.367, *P *= 0.005) were found in patients newly diagnosed with bipolar disorder, but not in their unaffected first-degree relatives compared with matched healthy individuals (data adjusted for sex and age). Most patients with bipolar disorder (94.7%) were diagnosed within the preceding 2 years, and the average illness duration, defined as time from first mood episode, was 10 years.

**Conclusion:**

Our findings of elevated prevalence of metabolic syndrome and insulin resistance in patients with newly diagnosed bipolar disorder highlight the importance of screening for these conditions at an early stage to employ adequate and early care reducing the risk of cardiovascular disease and premature death.

## Introduction

Bipolar disorder (BD) is associated with a decreased life expectancy of 8–12 years (Laursen et al. 2013; Kessing et al. 2015) with cardiovascular disease (CVD) being the leading cause of excess mortality (Osby et al. 2001). Patients with BD have increased risk of developing CVD compared with the general population (Goldstein 2017), and the American Heart Association recognizes BD in youth as a moderate-risk condition for early CVD and accelerated atherosclerosis (Goldstein et al. 2015). Metabolic syndrome (metS) constitutes a cluster of CVD risk factors consisting of abdominal obesity, diabetes and/or raised fasting plasma glucose, dyslipidemia and high blood pressure (Alberti et al. 2006). In general, presence of metS doubles the risk of CVD related mortality (Alberti et al. 2006). In patients with BD the prevalence of metS is doubled compared with the general population, an observation that appears to be a global phenomenon (Vancampfort et al. 2013).

Insulin resistance and central obesity are central components of metS (Alberti et al. 2006). Further, insulin resistance, metS and type 2 diabetes may separately be associated with adverse outcomes in patients with BD such as rapid cycling, treatment resistance to BD, and progression into a chronic illness course (Calkin et al. 2015; McIntyre et al. 2010; Cairns et al. 2018). Overweight and obesity have also been associated with increased severity of bipolar depression and a decreased treatment response that diminishes with increasing body mass index (BMI) (Kemp et al. 2010; Calkin et al. 2009). Similarly, the number and frequency of illness episodes seem to be increased in dysglycemic patients with BD compared with euglycemic patients (Calkin et al. 2015) and dysglycemia has been suggested to constitute a moderator of illness progression in BD (Mansur et al. 2016).

Despite robust evidence of the detrimental effects of metabolic comorbidity in BD, it is largely unknown whether risk factors for metS are present in newly diagnosed BD. Three prior case–control studies of metS in a small number of patients newly diagnosed with BD did not find a higher prevalence of metS compared with healthy individuals (Guha et al. 2014; Taylor et al. 2010; Wulsin et al. 2018). However, due to small sample sizes (n = 23–56) a true difference in prevalence may possibly have been overlooked. One of these studies (Guha et al. 2014), additionally, is the only case–control study of insulin resistance in patients newly diagnosed with BD, and they found higher insulin resistance index but not higher rates of metS in medication free, newly diagnosed patients with BD (Guha et al. 2014). However, the age of the participants (43.2 ± 10.6 years) suggested that the patients with BD were in a relatively late illness stage (Guha et al. 2014). Apart from one small study observing decreased high-density lipoprotein (HDL) cholesterol in first-degree unaffected relatives of patients with BD (Sobczak et al. 2004), it is largely unknown whether risk factors for metS, are present in unaffected first-degree relatives of BD.

## Aims of the study

The aims of the present study were: (I) to compare the prevalence of metS and levels of insulin resistance, as well as individual risk factors for metS and insulin resistance between patients with newly diagnosed BD and their unaffected first-degree relatives with healthy individuals without personal or first-degree family history of affective disorder; and (II) to determine to what extent illness and medication variables in patients with BD were associated with metS and insulin resistance.

We hypothesized that the prevalence of metS and insulin resistance was higher in patients with newly diagnosed BD and—to a lesser degree—in their unaffected first-degree relatives compared with healthy individuals without a family history of psychiatric disorders.

## Materials and methods

### Study design

The present report is a cross-sectional investigation of baseline data from the ongoing, longitudinal Bipolar Illness Onset Study (BIO), which aims to identify composite biomarkers for BD (Kessing et al. 2017). Participants were recruited from June 2015 to September 2017. The study protocol was approved by the Committee on Health Research Ethics of the Capital region of Denmark (protocol No. H-7-2014-007) and the Danish Data Protection Agency, Capital Region of Copenhagen (RHP-2015-023). Written informed consent was provided by all participants. The study complied with the Declaration of Helsinki principles (Seoul, October 2008).

### Participants

#### Patients with bipolar disorder

Patients were recruited from the Copenhagen Affective Disorder Clinic that covers the greater Copenhagen area of 1.6 million inhabitants (The Capital Region). All patients having a first episode of (hypo)mania or newly diagnosed with BD were routinely invited to participate in the study by the clinicians in the Copenhagen Affective Disorder Clinic (Kessing et al. 2017). Inclusion criteria were an ICD-10 diagnosis of a single manic episode or BD and age 15–70 years. Exclusion criteria included having an organic BD secondary to brain injury. The patients received treatment as usual without interference from study investigators.

#### Unaffected first-degree relatives

Siblings and children of the included patients with BD were invited to participate upon consent by the participating patient. Inclusion criteria were being a first-degree relative of an included patient with BD and age 15–40 years. Exclusion criteria included ICD-10 diagnoses of substance abuse, psychotic illnesses and mood disorders. We did not restrict the number of participating unaffected first-degree relatives per patient with BD as we adjusted for familial relationship in our analysis.

#### Healthy individuals

Age and sex-matched healthy individuals were recruited on random days among blood donors from the Blood Bank at Rigshospitalet, Copenhagen, Denmark. The inclusion criterion was age 15–70 years. Exclusion criteria were a personal or first-degree family history of psychiatric disorders that had required treatment.

### Clinical assessments

In the Copenhagen Affective Disorder Clinic, a specialist in psychiatry diagnosed patients with BD according to ICD-10 and classified them as type I or type II according to DSM-5 at the beginning of a two-year treatment program.

All participants were assessed by a medical doctor or a psychologist trained in diagnosing BD. The clinical diagnosis of BD was confirmed using the semi-structured interview: the Schedules for Clinical Assessment in Neuropsychiatry (SCAN) (Wing et al. 1990). Diagnosis of the current affective state was based on ICD-10 criteria. Severity of depressive and manic symptoms was assessed using the Hamilton Depression Rating Scale-17 items (HAMD-17) (Hamilton 1960) and the Young Mania Rating Scale (YMRS) (Young et al. 1978) respectively. Sleep quality was assessed using the Pittsburgh Sleep Quality Index (PSQI) (Buysse et al. 1989) and physical activity was assessed using the International Physical Activity Questionnaire (IPAQ) (Craig et al. 2003), which were both administered at the day of assessment. Medication, alcohol intake and smoking habits were recorded. Alcohol intake was stratified into low intake (0–4 units per week), moderate intake (5–13 units per week) and high intake (> 13 units per week).

Absence of lifetime psychiatric morbidity defined by ICD-10 was confirmed for healthy individuals whereas psychiatric morbidity of F34 and higher (e.g., cyclothymia, anxiety and personality disorders) according to ICD-10 were registered for unaffected relatives.

### Anthropometric assessment

After a 10-min rest blood pressure was measured in a sitting position using a calibrated automatic sphygmanometer (Microlife BP A3 plus) at the same day as the clinical assessment. Further, waist circumference was measured at the midpoint between the lowest rib and the iliac crest in an upright position to the nearest millimeter as described in the World Health Organisation’s guidelines (Cornier et al. 2011). Lightly dressed and without shoes, height was measured to the nearest millimeter on a rigid stadiometer and weight was measured to the nearest 0.1 kg using a calibrated floor scale (Kern MPE PM^®^).

### Laboratory methods

Fasting blood samples were collected in a resting state between 7.30 AM and 10 AM at the same day as the clinical assessment. Five milliliters of blood was drawn by venipuncture into an EDTA containing vacuum tube (Vacuette^®^) and within 30 min centrifuged at 1590 g and 4 °C for 15 min. Plasma was aliquoted into Eppendorf^®^ tubes and kept frozen at − 80 °C until P-Insulin was assayed. Plasma/serum concentrations of glucose, triglyceride, HbA1c and HDL cholesterol were measured using standard laboratory routine assays. Blood sampling and all aspects of laboratory processing were done at the Department of Clinical Biochemistry, Rigshospitalet, by laboratory specialists blinded with respect to participant status.

### Determination of metabolic syndrome, type 2 diabetes and insulin resistance

Metabolic syndrome was defined according to the International Diabetes Federation 2006 criteria (Alberti et al. 2006). Participants were considered having type 2 diabetes if they met at least one of the following three criteria: a diagnosis of type 2 diabetes, use of antidiabetic treatment (without other indication), and/or having a hemoglobin A1c (HbA1c) level of 48 mmol/mol or above. Insulin resistance was calculated from fasting plasma glucose and insulin using the homeostasis model assessment of insulin resistance (HOMA-IR) (HOMA-IR = (plasma glucose (mmol/L) × plasma insulin (mIU//L)/22.5 (Hermans et al. 1999)). To convert insulin from pmol/L to µIU/ml = mIU/L we divided with six, which has been found to be the most appropriate conversion factor (Larsen et al. 2017).

### Statistical analyses

Descriptive data were analyzed by Chi square tests for categorical data and by Student’s *t* test and Mann–Whitney U tests for two independent groups for continuous data, according to whether assumptions of normal distribution were met or not. Continuous data were presented as median and quartiles if assumptions of normal distribution were not met.

For illustrative purposes, we first compared prevalence of metS and levels of insulin resistance in an unadjusted generalized mixed effect model (categorical outcome) and a linear mixed effect model (continuous outcome), respectively, with familial relationship as random effect, to account for the correlation between family-related individuals. In a second model we adjusted for age and sex and as a third fully adjusted model we further added smoking status and alcohol intake as covariates.

In analyses among patients with BD we explored the associations between medication and illness characteristics and metS and insulin resistance, respectively, in separate models to retain sufficient statistical power. In these models, illness duration, BD type (I or II), current medication type in the form of lithium (yes/no), antipsychotics (yes/no), antidepressants (yes/no) and antiepileptics (yes/no) and, finally, receiving psychotropics with metabolic adverse effects were entered as predictors along with the covariates age and sex. All daily psychotropic medication except for lamotrigine and aripiprazole were considered psychotropics with metabolic adverse effects (Abosi et al. 2018). The category ‘not receiving psychotropics with metabolic adverse effect’ included patients not receiving psychotropic medication as well as patients receiving lamotrigine, aripiprazole or low dose quetiapine (i.e., not exceeding 50 mg daily). Illness duration was defined as time from first episode (depressive, hypomanic, manic or mixed episode). In a separate analysis we explored the association between demographic- and lifestyle variables and metS and insulin resistance among patients with BD, with these as dependent variables, and age, sex, smoking status, alcohol intake, sleep quality and physical activity as independent variables.

For all parametric tests, insulin resistance was transformed by the natural logarithm. Results are presented as back transformed values with a parameter estimate B, expressing the ratio between increments in independent variables. In the generalized linear mixed models, the model estimate, logit, was back transformed and presented as odds ratios. All model assumptions were met. The level of statistical significance was set at p < 0.05. Statistical analyses were performed using R version 3.3.2 and SPSS version 22.

## Results

### Demographic and clinical characteristics

We included 206 patients with BD, 50 of their unaffected relatives and 109 healthy individuals. Five patients with BD were Asian, five patients with BD were of mixed Asian and Caucasian ancestry and the remaining participants were Caucasian. Demographic and clinical variables of the study participants are presented in Table 1. Unaffected relatives were from 39 distinct families as 11 patients with BD had two of their unaffected relatives included. The patients with BD and healthy individuals were comparable in age (median [interquartile range] age 29.5 [24–37] vs. 28 [24–36.5] years*, P *= 0.8). Sex distribution in the three groups (patients with BD, unaffected relatives, healthy individuals) was similar. The unaffected first-degree relatives were younger than healthy individuals (*P *= 0.024). Healthy individuals had higher education level than patients with BD (*P *= 0.001), whereas education level did not differ between unaffected relatives and healthy individuals. Levels of activity, measured in metabolic equivalent minutes, were lower in patients with BD compared with healthy individuals (*P *= 0.013) and did not differ between unaffected relatives and healthy individuals (*P *= 0.9). More patients with BD and unaffected relatives reported sleep disturbances than healthy individuals (*P *< 0.001 and *P *= 0.001, respectively). Patients with BD had a lower weekly alcohol intake than healthy individuals (*P *< 0.001), whereas alcohol units per week were similar in unaffected relatives and healthy individuals (*P *= 0.1). Smoking was more common among patients with BD and their unaffected relatives compared to healthy individuals (Chi squared < 0.001). Among the patients with BD, 81.1% (n = 167) were diagnosed within the preceding year; additionally, 13.6% (n = 28) were diagnosed within the last 2 years, whereas the remaining 5.4% (n = 11) were diagnosed within the preceding 7 years.

**Table 1:** Demographic and clinical variables in patients with bipolar disorder (BD), their unaffected first-degree relatives (UR) and healthy individuals (HC)

	BD	UR	HC
N	206	50	109
Metabolic syndrome	31 (15)	3 (6)	6 (5.5)
Insulin resistance (HOMA-IR)	2.06 [1.44–3.25]	2.10 [1.59–2.63]	1.73 [1.35–2.42]
Age (years)	29.5 [24–37]	26.5 [22–31.3]	28 [24–36.5]
Sex (% female)	126 (61.2)	25 (50)	66 (60.6)
Education (years total)	15 [12.5–16]	15 [13–17]	15.5 [14.5–17]
Number of smokers (%)	95 (46.1)	13 (27.1)	10 (9.3)
Alcohol, units per week	2 [0–7]	2 [1–8]	5 [2–7]
Exercise (IPAQ, MET-minutes per week)	1680 [698–3051]	2373 [754–5270]	2040 [1160–4320]
Sleep disturbance (PSQI total score)	9 [6–13]	5.5 [3.8–8.3]	4 [2–6]
HDRS-17	9 [5–15]	2 [0–4]	1 [0–2]
YMRS	2 [0–6]	0 [0–2]	1 [0–2]
BD I	75 (34.4)	–	–
BD II	127 (61.7)	–	–
Single manic episode	4 (1.9)	–	–
Age of onset	17 [14–21]	–	–
Illness duration (years)	10 [6–16]	–	–
Untreated bipolar disorder (years)^a^	5 [1–11]	–	–
Manic episodes	0 [0–1]	–	–
Hypomanic episodes	4 [2–15]	–	–
Depressive episodes	6 [3–15]	–	–
Mixed episodes	0 [0–0]	–	–
Total affective episodes	12.5 [6–30]	–	–
Hospitalizations	0 [0–1]	–	–
*Current affective episode*			
Remission	124 (60.2)	–	–
Mild/moderate depressive episode	42 (20.4)	–	–
Severe depressive episode	6 (2.9)	–	–
Mixed episode	10 (4.9)	–	–
Hypomanic episode	17 (8.3)	–	–
Manic episode	2 (1)	–	–
N.A.	2 (1)	–	–
*Medication*			
No psychotropic medication	29 (14.1)	–	–
Lithium treatment	74 (35.9)	–	–
Antiepileptic treatment	108 (52.4)	–	–
Antidepressant treatment	55 (26.7)	–	–
Antipsychotic treatment	67 (32.5)	–	–
Psychotropic medication with metabolic adverse effects	127 (61.7)	–	–

Continuous variables are presented as median [interquartile range]. Categorical variables are presented as n (%). *HOMA-IR* the Homeostasis model assessment of insulin resistance, *IPAQ* International Physical Activity Questionnaire, *MET-minutes* metabolic equivalent minutes, *PSQI*: Pittsburgh Sleep Quality Index, *HAMD-17* 17-item Hamilton Depression Rating Scale, *YMRS* Young Mania Rating Scale, *BD I and BD II* Bipolar Disorder type I and II, respectively, *N.A*. not available

^a^Untreated bipolar disorder was defined as time from first manic, hypomanic or mixed episode to time of diagnosing bipolar disorder

### Metabolic syndrome in patients with bipolar disorder, their unaffected first-degree relatives and healthy individuals

**Fig. 1:**
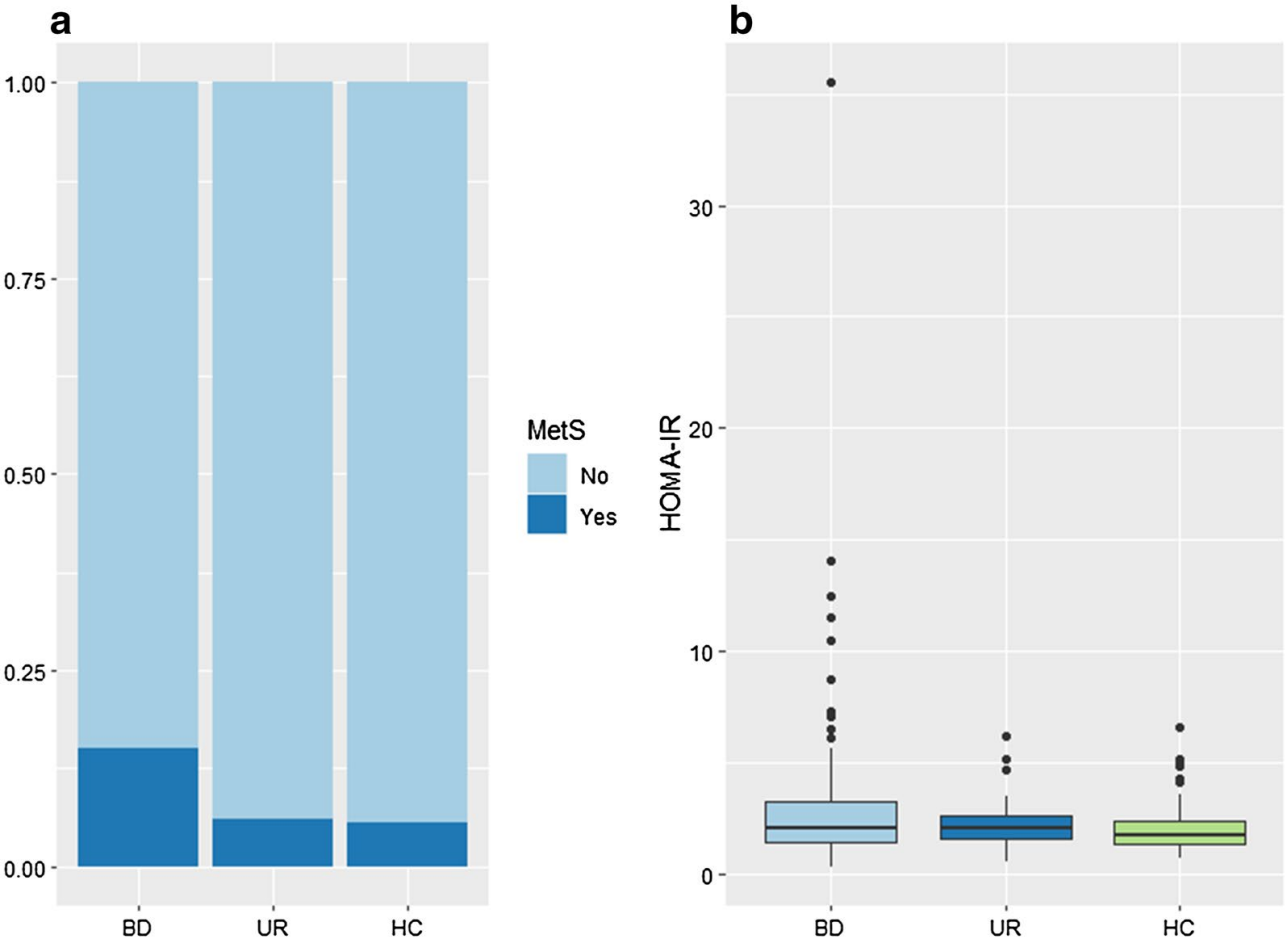
**a** Clustered bar chart of metabolic syndrome and **b** box plot of insulin resistance in patients with newly diagnosed bipolar disorder (BD), their unaffected first-degree relatives (UR) and healthy individuals (HC). The lower and upper hinges in the box plot correspond to the first and third quartiles and the upper and lower whiskers extend from the hinge to the largest and lower value, respectively, no further than 1.5 times the interquartile range from the hinge. Data beyond the end of the whiskers are plotted individually. MetS: metabolic syndrome; HOMA-IR: homeostasis model assessment of insulin resistance

As depicted in Fig. 1, 15.0% of patients with BD, 6.0% of their unaffected first-degree relatives and 5.5% of healthy individuals met criteria for metS. In the unadjusted analysis, the prevalence of metS was higher in patients with BD (B = 3.041, 95% CI 1.227–7.535, *P *= 0.016) but not in their unaffected relatives (B = 1.096, 95% CI 0.263–4.570, p = 0.9) compared with healthy individuals (Table 2, model 1a). Adjusted for age and sex, patients with BD had 3.5 higher risk of having metS than healthy individuals (B = 3.529, 95% CI 1.378–9.041, *P *= 0.009), whereas no statistically significant difference was found between unaffected relatives and healthy individuals (B = 1.472, 95% CI 0.334–6.497, *P *= 0.6) (Table 2, model 1b). In a fully adjusted model controlling additionally for smoking status and alcohol use, however, the observed difference in metS between the groups was no longer statistically significant (Table 2, model 1c). In a comparable model, there was no statistical difference in the prevalence of metS between unaffected relatives and patients with BD (B = 0.417, 95% CI 0.118–1.467, *P *= 0.2). Repeating the main analysis of metS between groups adjusted for age and sex, excluding the two patients with BD with type 2 diabetes, did not change the results (B = 3.250, 95% CI 1.270–8.319, *P *= 0.009).

**Table 2:** Metabolic syndrome and insulin resistance in patients with bipolar disorder (BD) and their unaffected first-degree relatives (UR) compared with healthy individuals (HC)

Model		B	95% CI	*P* value
1	Metabolic syndrome			
a	BD vs. HC	3.041	1.227–7.535	0.016
	UR vs. HC	1.096	0.263–4.570	0.9
b	Male vs. female sex	1.954	1.142–2.168	0.059
	Age	1.574	1.142–2.168	0.056
	BD vs. HC	3.529	1.378–9.041	0.009
	UR vs. HC	1.472	0.334–6.497	0.6
c	Male vs. female sex	2.117	0.998–4.494	0.051
	Age	1.580	1.137–2.197	0.006
	BD vs. HC	1.918	0.669–5.498	0.2
	UR vs. HC	1.108	0.027–0.432	0.9
	No smoking	0.332	0.151–0.730	0.006
	High vs. low alcohol intake	1.107	0.369–3.318	0.9
	Moderate vs. low alcohol intake	0.739	0.317–1.727	0.5
2	Insulin resistance			
a	BD vs. HC	1.203	1.058–1.367	0.005
	UR vs. HC	1.092	0.0910–1.311	0.3
b	Male vs. female sex	1.095	0.977–1.238	0.1
	Age	0.996	0.989–1.002	0.2
	BD vs. HC	1.203	1.059–1.367	0.005
	UR vs. HC	1.069	0.889–1.287	0.5
c	Male vs. female sex	1.129	1.001–1.273	0.048
	Age	0.995	0.982–1.002	0.2
	BD vs. HC	1.129	0.982–1.234	0.1
	UR vs. HC	1.019	0.842–1.234	0.8
	Smoking	1.113	0.974–1.271	0.1
	High vs. low alcohol intake	0.857	0.704–1.044	0.1
	Moderate vs. low alcohol intake	0.905	0.796–1.029	0.1

Model 1: Generalized linear mixed model with metabolic syndrome as the dependent variable. B represents odds ratios

Model 2: Linear mixed model with insulin resistance as the dependent variable. B represents back transformed beta values

### Insulin resistance in patients with bipolar disorder, their unaffected first-degree relatives and healthy individuals

Measurements of insulin were missing in three patients, one unaffected relative and three healthy individuals, consequently these seven participants were only included in analyses of metS but not of insulin resistance. As shown in Fig. 1 the median [interquartile range] of insulin resistance (HOMA-IR) was 2.06 [1.44–3.25] in patients with BD, 2.10 [1.59–2.63] in unaffected relatives and 1.73 [1.35–2.42] in healthy individuals. In the unadjusted model, levels of insulin resistance were 20.3% higher in patients with BD compared with healthy individuals (B = 1.203, 95% CI 1.058–1.367, *P *= 0.005) while no difference was found between unaffected relatives and healthy individuals (Table 2, model 2a). After adjusting for age and sex the levels of insulin resistance remained elevated in patients with BD compared with healthy individuals (B = 1.203, 95% CI 1.059–1.367, *P *= 0.005) (Table 2, model 2b). In a comparable model, there was no statistical difference in levels of insulin resistance between patients with BD and unaffected relatives (B = 1.125, 95% CI 0.958–1.321, *P *= 0.1). In a fully adjusted model controlling additionally for smoking status and alcohol use, however, the observed difference in insulin resistance between the groups was no longer statistically significant (Table 2, model 2c). In a post hoc analysis, we added waist circumference as a covariate in the fully adjusted model and it did not alter the findings, however, waist circumference was associated with insulin resistance (B = 1.023, 95% CI 1.018–1.028, *P* ≤ 0.001)).

We repeated our main analysis of insulin resistance adjusted for age and sex, excluding the two patients with BD with type 2 diabetes and this did not change the results (B = 1.199, 95% CI 1.055–1.362, *P *= 0.005).

### Individual components of the metabolic syndrome and insulin resistance

Levels of the individual components of the metS and insulin resistance, respectively, are shown in Table 3. Patients with BD had a larger waist circumference (*P *= 0.008) than healthy individuals and in explorative sex-specific analyses female patients with BD had a larger median [interquartile range] waist circumference than healthy female individuals (83 [76–90] cm vs. 78 [74–85] cm *P *= 0.014) whereas male patients with BD did not differ from healthy male individuals (91 [84–104] cm vs. 88 [82–94] cm *P *= 0.7). Compared with healthy individuals, patients with BD had higher levels of triglycerides (*P *< 0.001), fasting glucose (*P *= 0.047) and fasting insulin (*P *= 0.009), and lower levels of HDL cholesterol (*P *= 0.001) and systolic blood pressure (*P *= 0.004). Unaffected relatives had lower levels of HDL cholesterol than healthy individuals (*P *= 0.017), whereas the other components did not differ between these two groups.

**Table 3:** Individual components of the metabolic syndrome and insulin resistance in patients with bipolar disorder (BD) and their unaffected first-degree relatives (UR) compared with healthy individuals (HC)

	BD^1^	UR^2^	HC^3^	P-value
N	206	50	109	
Waist circumference (cm)	85 [78–94]	79 [74–89]	82 [75–90]	0.008^1−3^ 0.4^2−3^
Triglycerides (mmol/L)	0.94 [0.70–1.39]	0.77 [0.66–1.03]	0.76 [0.61–0.97]	< 0.001^1−3^ 0.4^2−3^
HDL cholesterol (mmol/L)	1.49 [1.24–1.83]	1.52 [1.29–1.79]	1.68 [1.35–1.94]	0.001^1−3^ 0.017^2−3^
Systolic blood pressure (mmHg)	120 [111–129]	124 [114–135]	124 [116–136]	0.004^1−3^ 0.9^2−3^
Diastolic blood pressure (mmHg)	77 [70–83]	78 [70–84]	77 [72–85]	0.1^1−3^ 0.3^2−3^
Fasting plasma glucose (mmol/L)	5.0 [4.8–5.4]	5.1 [4.8–5.4]	5.0 [4.7–5.2]	0.047^1−3^ 0.1^2−3^
Type 2 diabetes	2 (0.9)	0	0	
Insulin (pmol/L)	55.8 [39.7–84.8]	56.8 [42.0–69.9]	48.9 [38.5–63.6]	0.009^1−3^ 0.1^2−3^

*HDL* high density lipoprotein, *MmHg* millimeters of mercury. Continuous variables are presented as median [interquartile range]

### Association between illness- and medication variables and metabolic syndrome and insulin resistance among patients with bipolar disorder

Illness duration (B = 0.637, 95% CI 0.409–0.998, *P *= 0.047) was negatively associated with metS (Table 4, model 1). No association between individual psychotropic medication types and metS was observed. Lithium treatment was positively associated with insulin resistance (B = 1.242, 95% CI 1.045–1.478, *P *= 0.014), while no association was observed between illness duration or other individual types of psychotropic medication and insulin resistance (Table 4, model 2). When grouping medication according to their likely metabolic impact, treatment with psychotropic medication with metabolic adverse effects was associated with higher insulin resistance (B = 1.298, 95% CI 1.088–1.550, *P *= 0.004). However, no association was found between treatment with psychotropic medication with metabolic adverse effects and metS (B = 2.325, 95% CI 0.873–6.194, *P *= 0.09). No association was found between BD subtype and metS or insulin resistance.

**Table 4:** Metabolic syndrome and insulin resistance in patients with bipolar disorder

	Model	B	95% CI	P-value
1	Metabolic syndrome			
a	Illness duration^a^	0.637	0.409–0.993	0.047
b	BD type I vs. type II	0.769	0.345–1.705	0.5
c	Lithium	0.993	0.438–2.252	1.0
d	Antipsychotics	0.845	0.361–1.981	0.7
e	Antiepileptics	0.782	0.350–1.748	0.5
f	Antidepressants	2.517	1.717–3.691	0.7
g	Medication with metabolic adverse effects	2.325	0.873–6.194	0.1
2	Insulin resistance			
a	Illness duration^a^	0.987	0.974–1.000	0.056
b	BD type I vs. type II	0.891	0.745–1.065	0.2
c	Lithium	1.242	1.045–1.478	0.014
d	Antipsychotics	1.082	0.903–1.294	0.4
e	Antiepileptics	0.874	0.736–1.040	0.1
f	Antidepressants	1.075	0.882–1.310	0.3
g	Medication with metabolic adverse effects	1.298	1.088–1.550	0.004

Model 1: Separate generalized linear mixed models of metabolic syndrome (a–g) in patients with BD, adjusted for age and sex. B represents odds ratios

Model 2: Separate linear mixed models of insulin resistance (a–g) in patients with BD, adjusted for age and sex. B represents back transformed beta values

BD I and BD II: Bipolar Disorder type I and II, respectively. ^a^Illness duration was defined as time from first episode (depressive, hypomanic, manic or mixed episode)

### Association between demographic- and lifestyle variables and metabolic syndrome and insulin resistance among patients with bipolar disorder

There was no statistically significant association between metS and sex, smoking status, alcohol intake, sleep quality or physical activity among patients with BD. A moderate alcohol intake was associated with lower insulin resistance compared with a low alcohol intake in patients with BD (B = 0.868, 95% CI 0.763–0.988, *P *= 0.033) and male sex was associated with higher insulin resistance (B = 1.174, 95% CI 1.037–1.328, *P *= 0.011). Smoking status, sleep quality and physical activity were not associated with insulin resistance in patients with BD.

## Discussion

### Main results

We found a higher prevalence of metS and greater degree of insulin resistance in 206 newly diagnosed bipolar patients with a median age of 29.5 [24–37] years, but not in the 50 unaffected first-degree relatives, compared with 109 age and sex-matched healthy individuals. Considering smoking status and alcohol use in an adjusted model, however, the observed difference between patients with bipolar disorder and healthy individuals was no longer statistically significant. Comparing the individual components of the metS and insulin resistance between groups revealed an adverse metabolic profile among patients with newly diagnosed BD who had a larger waist circumference, more dyslipidemia with higher levels of triglycerides and lower levels of HDL cholesterol and higher levels of fasting glucose and insulin compared with healthy individuals. The unaffected relatives had lower levels of HDL cholesterol than healthy individuals. In analyses among patients with BD, treatment with lithium as well as treatment with medication with adverse metabolic effects were associated with higher levels of insulin resistance, whereas illness duration was negatively associated with metS.

### Comparison to other case–control studies in patients with newly diagnosed bipolar disorder

Three previous studies investigated metS in patients with newly diagnosed BD compared with healthy individuals (Guha et al. 2014; Taylor et al. 2010; Wulsin et al. 2018) and one of these additionally assessed insulin resistance (Guha et al. 2014). Unlike our study, these prior studies did not include analyses adjusted for alcohol and smoking. Two of these studies found no difference in prevalence of metS in patients newly diagnosed with BD compared with healthy individuals (Guha et al. 2014; Taylor et al. 2010). However, in the study by Guha et al.(2014) including 55 patients with BD and 25 controls, the high mean age 43.2 years (SD: 10.6) of patients with BD indicated that patients were possibly in a later illness stage compared with our population of newly diagnosed patients with BD. Furthermore, their population of patients with BD and healthy individuals had more central obesity than participants in our study sample. The study by Taylor et al. (2010) included only 24 newly diagnosed patients with BD, and may have been underpowered to detect differences between groups. The third case–control study (Wulsin et al. 2018) found comparable rates of metS to our findings and the lack of statistically significance may have been due to lack of power owing to a relatively small group of healthy individuals (n = 56). Further, these three studies of metS in patients with newly diagnosed BD all assessed metS using the National Cholesterol Education Program adult treatment panel III (ATPIII) definition, whereas we used the International Diabetes Federation’s criteria, which are the most commonly used criteria in Europe with cut-offs for central obesity specific for populations of Europid origin. The ATPIII definition can be met without having central obesity and the cut-off for central obesity for populations of North American origin is more liberal than the International Diabetes Federation’s criteria (Kaur 2014). If these studies had used the International Diabetes Federation’s criteria it is possible that they had found similar results to ours (e.g. in the study by Guha et al. (2014) only seven out of 55 patients newly diagnosed with BD fulfilled the ATPIII criteria). Similarly to the results by Guha et al. (2014) we found elevated levels of insulin resistance in patients with newly diagnosed BD, although as discussed above, patients with BD in our study were substantially younger than patients with BD in the study by Guha et al. (2014).

### Illness- and medication variables

In patients with BD we observed a negative association between illness duration and metS while extant research appears to have indicated a lack of such an association (Vancampfort et al. 2013). However, the confidence interval for the estimate of the association was wide and although we adjusted the analysis for age and sex it cannot be excluded that the finding is due to residual confounding. We did not replicate previous findings of treatment with antipsychotics being associated with increased prevalence of metS (Vancampfort et al. 2013), but found treatment with lithium associated with increased levels of insulin resistance. However, the observed effect was rather small and due to the observational design and the limitations in estimating effects of single treatment categories in patients receiving several types of medications, caution should be observed when interpreting this finding. Given those limitations, it may be more meaningful to consider medication in their capacity to induce metabolic changes, when estimating the association between medication and metS or insulin resistance. Using this approach, we found that patients using medication with metabolic side effects had more insulin resistance, but not a higher risk of metS, compared with patients that did not use such medication. The association between medication with capacity to induce metabolic changes and metS did not reach statistically significance, however, this may be due to the relatively low power of the analysis, and overall, our results point to medication as an important risk factor for metS.

In accordance with Calkin et al.’s (2015) findings we did not find association between the type of BD and insulin resistance. Finally, the type of BD was neither associated with metS.

### Individual components of the metabolic syndrome and insulin resistance

Patients with BD had larger waist circumferences than healthy individuals. Robust epidemiological evidence associates central obesity with increased overall mortality as well as excess death caused by CVD (Cornier et al. 2011; Pischon et al. 2008) and, notably, the median waist circumference of 83 cm in our female patients with BD exceeded the cut-off for central obesity defined by the International Diabetes Federation (≥ 80 cm) (Alberti et al. 2006), while waist circumference medians of male patients with BD and healthy individuals of both sexes were below the threshold for central obesity. We also found an adverse lipid profile in patients with BD with lower levels of HDL cholesterol and higher levels of triglycerides similar to recent findings in patients with BD compared with healthy individuals (Wulsin et al. 2018). The only individual component of metS not differing in patients with BD compared with healthy individuals was the systolic blood pressure which align with findings of a recent meta-analysis of metS and severe mental illness, where the risk of metS and all its individual components except for hypertension were elevated in patients with BD (Vancampfort et al. 2015).

Fasting insulin levels were higher in patients with BD compared with healthy individuals, supporting the findings by Guha et al. (2014). In contrast to that study, we also detected higher levels of fasting glucose in patients with newly diagnosed BD compared with healthy individuals, although the levels were within normal ranges in all three groups. Only mild non medicated type 2 diabetes is allowed among blood donors and we therefore repeated our main analyses of metS and insulin resistance excluding the two patients with BD with comorbid type 2 diabetes. This, however, did not alter the results of our main analyses, which we find to be most correct, as the two patients with BD were not in treatment for type 2 diabetes and were unaware of having type 2 diabetes at time of assessment, thus not treated, which could happen for blood donors as well.

### Possible predictors of metabolic syndrome and insulin resistance in patients with bipolar disorder

In the current study, patients with BD differed further from healthy individuals in regard to active smoking (Sun et al. 2012), alcohol intake (Alkerwi et al. 2009), activity level (Zhang et al. 2017) and sleep disturbances (Pulido-Arjona et al. 2018), which are major predictors of metS and insulin resistance. We found that 46% of the patients with BD were smokers, which is more than twice the prevalence of the Danish general population (SST 2017) versus 27% of their unaffected relatives and 9% of their healthy individuals. Smoking cessation increases the levels of HDL cholesterol (März et al. 2017) and has been shown to reduce the risk of metS (Sun et al. 2012). We found smoking to be associated with metS in our overall analyses (Sun et al. 2012), whereas we found no effect of activity level and sleep disturbances in patients with bipolar disorder. Further, in these models, we did not find associations between insulin resistance level and smoking, activity level and sleep disturbances, respectively. However, interpretation of these analyses should be made with caution as we are comparing relatively small subsamples of patients with BD. In the present study, patients with newly diagnosed BD had a lower alcohol intake than healthy individuals, and whereas alcohol in small amounts seems protective of metS high alcohol intake increases the risk of metS (Alkerwi et al. 2009). We did not find an association between metS and alcohol neither in comparison of the three groups or within patients with BD. However, alcohol intake was relatively low in all three groups in the present study suggesting a minor role of alcohol in the current observations. Our finding of higher insulin resistance level among patients with low alcohol intake compared with patients with moderate alcohol intake was unexpected. However, many patients with BD have had an increased alcohol intake the preceding months before initiating treatment in the Copenhagen Affective Disorder Clinic and were encouraged to restrict or completely avoid alcohol for the initial 3 months in the clinic. A preceding large alcohol intake would not be captured in our measure, thus our finding of a negative association between higher alcohol intake and lower prevalence of insulin resistance among patients with BD, should be interpreted with caution. Patients with BD were less physically active than healthy individuals. This may contribute to the present findings and could be due to symptoms related to BD but may also be related to the use of sedative medication. The lower level of activity could likely contribute to the lower levels of HDL cholesterol observed in the present study, such as observed in other studies of patients with BD (Chen et al. 2017). Finally, sleep disturbances in young adults are associated with dysmetabolism such as higher fasting glucose levels and triglyceride levels and lower levels of HDL cholesterol (Pulido-Arjona et al. 2018) and were more prevalent in patients with BD and in their unaffected first-degree relatives in the current study.

### Metabolic syndrome and insulin resistance in unaffected first-degree relatives

Studying unaffected relatives may help to clarify if there is a common shared pathophysiology of BD and type 2 diabetes and/or CVD (de Melo et al. 2017; SayuriYamagata et al. 2017). Apart from lower levels of HDL cholesterol in unaffected relatives compared with healthy individuals, our findings did not indicate dysmetabolism before onset of BD as levels of insulin resistance and rates of metS and their individual components did not differ from healthy individuals. Nonetheless, patients with BD and their unaffected first-degree relatives neither differ in prevalence of metS nor in levels of insulin resistance from patients with BD, thus we may possibly have overseen a true difference between our modest sample size of unaffected relatives and healthy individuals. Unaffected first-degree relatives had smoking prevalence at the same level as the Danish general population (SST 2017) and with respect to metS, where smoking is known to be a major predictor (Sun et al. 2012), the unaffected relatives could possibly be representative of the general population. Nonetheless, whereas the healthy individuals possibly were super healthy indicated by higher education- and activity level than the patients with BD and lower smoking prevalence than the Danish general population, the unaffected first-degree relatives may possibly be less healthy than the general population due to their psychiatric dispositions and consequently would not be a valid control group.

### Strengths and limitations

Advantages of the study include thoroughly assessment of a relatively large group of well-characterized patients with newly diagnosed BD and a group of their unaffected first-degree relatives. Further, our study profited from a high degree of standardization (e.g., blood sampling time, collection and analyses) at the same day as clinical and psychiatric evaluation. Most of our patients with BD (81.1%) were diagnosed within the preceding year and the maximum time with diagnosis was 7 years. The average delay in diagnosis of 5 years and illness duration (time from first mood episode) of 10 years accords with prior findings (Kessler et al. 2007; Baldessarini et al. 2007) and further illustrates the challenge of recruiting patients early in their course of illness due to the known diagnostic delay (Baldessarini et al. 2007).

Several limitations of our study should be considered. First, the sample size of unaffected relatives was moderate (n = 50) due to three major reasons; (1) missing consent from patients to contact their relatives, (2) unaffected relatives having a major psychiatric illness themselves and (3) lack of unaffected first-degree relatives satisfying the age range criteria (15–40 years). Due to this limited sample of healthy relatives, we were not able to include the measurements of sleep disturbances and physical activity in our fully adjusted analyses comparing metS and insulin resistance between groups. However, since there were no statistically significant differences between groups in the models including smoking status and alcohol intake, we find it unlikely, that the addition of sleep disturbances and physical activity measurements would have changed the results. Second, due to the cross-sectional study design, we cannot draw final conclusions regarding dysmetabolism before BD based on the findings in unaffected relatives of patients with BD, as the majority proportion of these will not develop BD. Currently we are expanding our study cohort in the longitudinal BIO study (Kessing et al. 2017), and following participants prospectively, including through onset of illness, will enhance our knowledge about dysmetabolism before onset of BD. Third, in contrast to well-established evidence we did not find association between antipsychotics and metS and insulin resistance, respectively (Vancampfort et al. 2013; Correll et al. 2008; Burghardt et al. 2018), however, we found an association between receiving psychotropics with metabolic adverse effects and insulin resistance. Further, lithium was also observed associated with insulin resistance. Caution in interpreting these findings should be made due to our categorization of treatment. Thus, our use of dichotomous treatment categories does not account for neither medication dose nor duration of treatment. Further, the concurrent treatment with multiple medication types reduces or may even eliminate the possibility to identify an isolated effect of one treatment category. We may therefore not fully capture the true effect of psychotropic medication on metabolism in the present study. However, using the variable of receiving psychotropics with metabolic adverse effects may possibly yield the most valid analysis of psychotropic medication in the present study. Nonetheless, our findings should be interpreted with caution and we cannot exclude the possibility that receiving psychotropics with metabolic adverse effects is the main driver of the identified higher metS prevalence and insulin resistance level in patients with BD compared with heatlhy individuals. Fourth, we included blood donors recruited in the Blood Bank at Rigshospitalet as healthy individuals. Blood donors may in some respects be regarded as super healthy individuals, and in our sample they had a longer education level, which is known to increase overall health (Golding et al. 2013) and they had a lower prevalence of smoking than the general population (SST 2017). Contrary to our expectations, the healthy individuals had higher systolic blood pressure and a higher alcohol intake than the patients with BD.

## Conclusions

Our findings of higher prevalence of metS and elevated insulin resistance levels in patients with newly diagnosed BD compared with healthy individuals highlight the pivotal role of early individualized prevention strategies targeted against smoking, overweight, dyslipidemia and dysglycemia in this population. Notably, smoking was highly prevalent in patients with newly diagnosed BD and—to a lesser degree—in their unaffected first-degree relatives. The well-established connection between smoking and metS indicate that smoking cessation is highly relevant in these populations. Further, our findings suggest that the high prevalence of smoking in our population of patients with BD was an important driver of the identified higher risk of metS. Similarly, central obesity was strongly associated with insulin resistance in patients with BD and found specifically in female patients newly diagnosed with BD. The major CVD risk accompanying central obesity further highlight the importance of maintaining a healthy fat distribution and intervening against central obesity from time of diagnosing BD. Preventing the development of metS, type 2 diabetes and CVD may diminish the risk of premature death and a chronic, disabling illness course in patients with BD. Clinically, it should be considered to include assessment of metS and insulin resistance aiming to prevent or identify metabolic diseases in patients with BD from the time of diagnosis.

## Significant outcomes

- Patients with newly diagnosed bipolar disorder but not their unaffected first-degree relatives had higher rates of metabolic syndrome and insulin resistance compared with healthy individuals.
- A larger waist circumference, more dyslipidemia and higher levels of fasting glucose and insulin were observed in patients with bipolar disorder compared with matched healthy individuals.
- Assessment of the individual components of the metabolic syndrome, insulin resistance and smoking from time of diagnosis should be considered to initiate adequate and early care reducing the risk of cardiovascular disease and premature death.

## Limitations

- The sample size of unaffected first-degree relatives was modest.
- Our categorization of psychotropic medication variables may not capture the true effect of psychotropic medication.

## Abbreviations

BD: bipolar disorder
metS: metabolic syndrome
BMI: body mass index
HDL: high-density lipoprotein
HOMA-IR: homeostasis model assessment of insulin resistance

## References

[CR1] Abosi O, Lopes S, Schmitz S, Fiedorowicz JG (2018). Cardiometabolic effects of psychotropic medications. Horm Mol Biol Clin Investig.

[CR2] Alberti G, Zimmet P, Shaw J, M. Grundy SM. IDF Consensus Worldwide Definition of the Metabolic Syndrome. 2006.

[CR3] Alkerwi A, Boutsen M, Vaillant M, Barre J, Lair ML, Albert A (2009). Alcohol consumption and the prevalence of metabolic syndrome: a meta-analysis of observational studies. Atherosclerosis.

[CR4] Baldessarini RJ, Tondo L, Baethge CJ, Lepri B, Bratti IM (2007). Effects of treatment latency on response to maintenance treatment in manic-depressive disorders. Bipolar Disord.

[CR5] Burghardt KJ, Seyoum B, Mallisho A, Burghardt PR, Kowluru RA, Yi Z (2018). Atypical antipsychotics, insulin resistance and weight; a meta-analysis of healthy volunteer studies. Prog Neuropsychopharmacol Biol Psychiatry.

[CR6] Buysse DJ, Reynolds CF, Monk TH, Berman SR, Kupfer DJ (1989). The Pittsburgh Sleep Quality Index: a new instrument for psychiatric practice and research. Psychiatry Res.

[CR7] Cairns K, McCarvill T, Ruzickova M, Calkin CV (2018). Course of bipolar illness worsens after onset of insulin resistance. J Psychiatr Res.

[CR8] Calkin C, van de Velde C, Ruzickova M, Slaney C, Garnham J, Hajek T (2009). Can body mass index help predict outcome in patients with bipolar disorder?. Bipolar Disord.

[CR9] Calkin CV, Ruzickova M, Uher R, Hajek T, Slaney CM, Garnham JS (2015). Insulin resistance and outcome in bipolar disorder. Br J Psychiatry.

[CR10] Chen YC, Tsai JC, Liou YM, Chan P (2017). Effectiveness of endurance exercise training in patients with coronary artery disease: a meta-analysis of randomised controlled trials. Eur J Cardiovasc Nurs.

[CR11] Cornier MA, Despres JP, Davis N, Grossniklaus DA, Klein S, Lamarche B (2011). Assessing adiposity: a scientific statement from the American Heart Association. Circulation.

[CR12] Correll CU, Frederickson AM, Kane JM, Manu P (2008). Equally increased risk for metabolic syndrome in patients with bipolar disorder and schizophrenia treated with second-generation antipsychotics. Bipolar Disord.

[CR13] Craig CL, Marshall AL, Sjostrom M, Bauman AE, Booth ML, Ainsworth BE (2003). International physical activity questionnaire: 12-country reliability and validity. Med Sci Sports Exerc.

[CR14] de Melo LGP, Nunes SOV, Anderson G, Vargas HO, Barbosa DS, Galecki P (2017). Shared metabolic and immune-inflammatory, oxidative and nitrosative stress pathways in the metabolic syndrome and mood disorders. Prog Neuropsychopharmacol Biol Psychiatry.

[CR15] SST. 2018. https://www.sst.dk/da/udgivelser/2018/~/media/D76CC353723F458E9F978B6BE1BF1BBC.ashx. Accessed 31 Jan 2019.

[CR16] Golding J, Northstone K, Miller LL, Davey Smith G, Pembrey M (2013). Differences between blood donors and a population sample: implications for case-control studies. Int J Epidemiol.

[CR17] Goldstein BI (2017). Bipolar disorder and the vascular system: mechanisms and new prevention opportunities. Can J Cardiol.

[CR18] Goldstein BI, Carnethon MR, Matthews KA, McIntyre RS, Miller GE, Raghuveer G (2015). Major depressive disorder and bipolar disorder predispose youth to accelerated atherosclerosis and early cardiovascular disease: a scientific statement from the American Heart Association. Circulation.

[CR19] Guha P, Bhowmick K, Mazumder P, Ghosal M, Chakraborty I, Burman P (2014). Assessment of insulin resistance and metabolic syndrome in drug naive patients of bipolar disorder. Indian J Clin Biochem.

[CR20] Hamilton M (1960). A rating scale for depression. J Neurol Neurosurg Psychiatry.

[CR21] Hermans MP, Levy JC, Morris RJ, Turner RC (1999). Comparison of insulin sensitivity tests across a range of glucose tolerance from normal to diabetes. Diabetologia.

[CR22] Kaur J (2014). A comprehensive review on metabolic syndrome. Cardiol Res Pract.

[CR23] Kemp DE, Gao K, Chan PK, Ganocy SJ, Findling RL, Calabrese JR (2010). Medical comorbidity in bipolar disorder: relationship between illnesses of the endocrine/metabolic system and treatment outcome. Bipolar Disord.

[CR24] Kessing LV, Vradi E, Andersen PK (2015). Life expectancy in bipolar disorder. Bipolar Disord.

[CR25] Kessing LV, Munkholm K, Faurholt-Jepsen J, Miskowiak KW, Nielsen LB, Frikke-Schmidt R, Ekstrøm C, Winther O, Pedersen BK, Poulsen HE, McIntyre RS, Kapczinski F, Gattaz WF, Bardram J, Mads F, Mayora O, Knudsen GM, Phillips M, Vinberg M (2017). The Bipolar illness onset study—research protocol for the BIO cohort study. BMJ Open.

[CR26] Kessler RC, Angermeyer M, Anthony JC, De Graaf RO, Demyttenaere K, Gasquet I (2007). Lifetime prevalence and age-of-onset distributions of mental disorders in the World Health Organization’s World Mental Health Survey Initiative. World Psychiatry.

[CR27] Larsen PB, Linneberg A, Hansen T, Friis-Hansen L (2017). Reference intervals for C-peptide and insulin derived from a general adult Danish population. Clin Biochem.

[CR28] Laursen TM, Wahlbeck K, Hallgren J, Westman J, Osby U, Alinaghizadeh H (2013). Life expectancy and death by diseases of the circulatory system in patients with bipolar disorder or schizophrenia in the Nordic countries. PLoS ONE.

[CR29] Mansur RB, Rizzo LB, Santos CM, Asevedo E, Cunha GR, Noto MN (2016). Impaired glucose metabolism moderates the course of illness in bipolar disorder. J Affect Disord.

[CR30] März W, Kleber ME, Scharnagl H, Speer T, Zewinger S, Ritsch A (2017). HDL cholesterol: reappraisal of its clinical relevance. Clin Res Cardiol.

[CR31] McIntyre RS, Danilewitz M, Liauw SS, Kemp DE, Nguyen HT, Kahn LS (2010). Bipolar disorder and metabolic syndrome: an international perspective. J Affect Disord.

[CR32] Osby U, Brandt L, Correia N, Ekbom A, Sparen P (2001). Excess mortality in bipolar and unipolar disorder in Sweden. Arch Gen Psychiatry.

[CR33] Pischon T, Boeing H, Hoffmann K, Bergmann M, Schulze MB, Overvad K (2008). General and abdominal adiposity and risk of death in Europe. N Engl J Med.

[CR34] Pulido-Arjona L, Correa-Bautista JE, Agostinis-Sobrinho C, Mota J, Santos R, Correa-Rodriguez M (2018). Role of sleep duration and sleep-related problems in the metabolic syndrome among children and adolescents. Ital J Pediatr.

[CR35] SayuriYamagata A, Brietzke E, Rosenblat JD, Kakar R, McIntyre RS (2017). Medical comorbidity in bipolar disorder: the link with metabolic-inflammatory systems. J Affect Disord.

[CR36] Sobczak S, Honig A, Christophe A, Maes M, Helsdingen RW, De Vriese SA (2004). Lower high-density lipoprotein cholesterol and increased omega-6 polyunsaturated fatty acids in first-degree relatives of bipolar patients. Psychol Med.

[CR37] Sun K, Liu J, Ning G (2012). Active smoking and risk of metabolic syndrome: a meta-analysis of prospective studies. PLoS ONE.

[CR38] Taylor V, McKinnon MC, Macdonald K, Jaswal G, Macqueen GM (2010). Adults with mood disorders have an increased risk profile for cardiovascular disease within the first 2 years of treatment. Can J Psychiatry.

[CR39] Vancampfort D, Vansteelandt K, Correll CU, Mitchell AJ, De Herdt A, Sienaert P (2013). Metabolic syndrome and metabolic abnormalities in bipolar disorder: a meta-analysis of prevalence rates and moderators. Am J Psychiatry.

[CR40] Vancampfort D, Stubbs B, Mitchell AJ, De Hert M, Wampers M, Ward PB (2015). Risk of metabolic syndrome and its components in people with schizophrenia and related psychotic disorders, bipolar disorder and major depressive disorder: a systematic review and meta-analysis. World Psychiatry.

[CR41] Wing JK, Babor T, Brugha T, Burke J, Cooper JE, Giel R (1990). SCAN. Schedules for clinical assessment in neuropsychiatry. Arch Gen Psychiatry.

[CR42] Wulsin LR, Blom TJ, Durling M, Welge JA, DelBello MP, Adler CM (2018). Cardiometabolic risks and omega-3 index in recent-onset bipolar I disorder. Bipolar Disord.

[CR43] Young RC, Biggs JT, Ziegler VE, Meyer DA (1978). A rating scale for mania: reliability, validity and sensitivity. Br J Psychiatry.

[CR44] Zhang D, Liu X, Liu Y, Sun X, Wang B, Ren Y (2017). Leisure-time physical activity and incident metabolic syndrome: a systematic review and dose-response meta-analysis of cohort studies. Metabolism.

